# Examining the impact of racial disparities on *Clostridioides difficile* infection outcomes at a Southern California academic teaching hospital

**DOI:** 10.1017/ice.2023.84

**Published:** 2023-11

**Authors:** Jina M. Lee, Anna Y. Zhou, Natalie M. Ortiz-Gratacos, Almas Al Isso, Karen K. Tan, Jacinda C. Abdul-Mutakabbir

**Affiliations:** 1 Department of Pharmacy, Loma Linda University Medical Center, Loma Linda, California; 2 Department of Pharmacy Practice, Loma Linda University School of Pharmacy, Loma Linda, California; 3 Division of Clinical Pharmacy, Skaggs School of Pharmacy and Pharmaceutical Sciences, University of California San Diego, La Jolla, California; 4 Division of the Black Diaspora and African American Studies, University of California San Diego, La Jolla, California

## Abstract

Racial differences in *Clostridioides difficile* infection (CDI) outcomes have been reported. In this study, minoritized patients with CDIs had prolonged hospitalizations and increased intensive care unit admissions. Chronic kidney disease was shown to partially mediate the relationship between race or ethnicity and severe CDI. Our findings suggest potential areas for equitable interventions.

Infectious diseases are major contributors to racial disparities in mortality within the United States, second only to cardiovascular diseases.^1^ Racial inequities stemming from longstanding structural and institutional racism have led to disparities in social determinants of health (eg, housing, socioeconomic status, and access to quality health services) and poor health outcomes for racially and ethnically minoritized patients (REM).^2^

*Clostridioides difficile* infection (CDI), a common hospital and community-acquired infection, is associated with prolonged hospitalizations and mortality, with reports of racial differences in patient outcomes.^3,4^ The Infectious Diseases Society of America (IDSA) has previously defined key risk factors for acquiring CDI;^5^ nonetheless, recent data have shown a linkage between pre-existing comorbidities (including chronic kidney disease or CKD) and severe or fulminant CDI.^6^ REM patients have been diagnosed with CKD more frequently than non–racially and ethnically minoritized (n-REM) individuals;^7^ however, research that explores the association between race or ethnicity and CKD (along with social determinants of health) in CDI outcomes is lacking.

In this study, we investigated racial differences in CDI clinical outcomes of hospitalized adults in a large, academic, minority-serving, medical center in Southern California.

## Methods

### Study design

This retrospective, descriptive study was approved by the Institutional Review Board of Loma Linda University Medical Center (LLUMC). Patients aged ≥18 years hospitalized with initial episodes of hospital or community-acquired CDI from January 2020 to June 2021 were screened for inclusion. Initial episodes were identified by *International Classification of Disease, Tenth Revision* (ICD-10) code A04.72 and included a positive test for *C. difficile*, defined as a positive assay for both GDH and *C. difficile* toxin, or a positive assay for either GDH or *C. difficile* toxin with a confirmatory positive PCR test. We applied the following exclusion criteria: patients who received CDI therapy initiated by a provider outside LLUMC, patients with recurrent CDI, patients discharged before therapy initiation, patients who did not have documented race or ethnicity, and patients who were pregnant.

For all included patients, medical records were reviewed for demographics, comorbid conditions, and insurance statuses. Potential risk factors for acquiring CDI were also collected. Patient race and ethnicity were self-reported. Ethnicity was categorized into “Hispanic or Latino” or “not Hispanic or Latino.” Patients were dichotomized by race and ethnicity into either the racially and ethnically minoritized (REM) group (ie, Hispanic, Black or African-American, Asian, Pacific Islanders, and patients from multiple ethnic backgrounds) or the non–racially and ethnically minoritized (n-REM), representing non-Hispanic White individuals. Data were managed using REDCap electronic data capture tools hosted at LLUMC.

### Outcome measures

The primary objective of this study was to compare the CDI outcomes between REM and n-REM groups. Outcomes collected include CDI disease severity as defined by the 2017 IDSA/Society for Healthcare Epidemiology of America (SHEA) *Clostridioides difficile* Clinical Practice Guideline,^5^ all-cause mortality during admission, length of stay (LOS), rate of all-cause intensive care unit (ICU) admission, ICU LOS, recurrence rate, and receipt of infectious diseases (ID) or gastrointestinal (GI) consult.

### Statistical analysis

The Pearson χ^2^ test for heterogeneity was used for categorical data. The Shapiro-Wilk test of normality was used for continuous variables. Parametric data were compared using the Student *t* test, and nonparametric data were compared using the Mann-Whitney *U* test. *P* ≤ .05 was considered as statistically significant.

We used counterfactual mediation analysis to examine the contribution of CKD to the association between race or ethnicity and severe or fulminant CDI, while controlling for age. The hypothesized causal structure is shown as a directed acyclic graph in Supplementary Fig. 1. We derived adjusted odds ratios for the direct effect (ie, the race or ethnicity association with severe or fulminant CDI not due to the mediator CKD), indirect effect (ie, the association due to the mediator CKD), and total effect (ie, direct and indirect effects). We calculated the percentage of the total effect that was mediated by CKD as follows:

[direct effect × (indirect effect – 1)/(total effect – 1)] × 100.

Statistical analyses were performed on SPSS Statistics version 26 software (IBM, Armonk, NY) and SAS version 9.4 software (SAS Institute, Cary, NC).

## Results

### Baseline characteristics

**Fig. 1.**
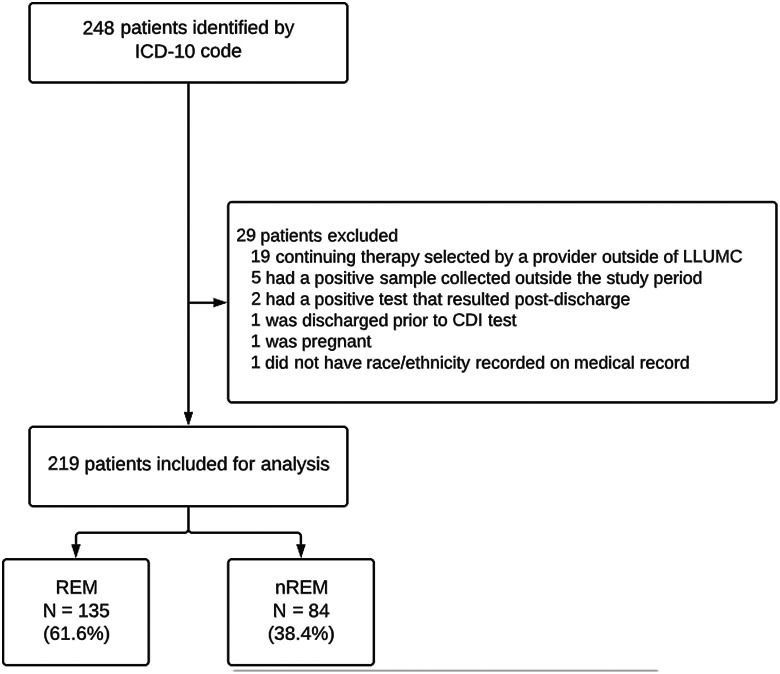
Patient baseline characteristics for inclusion and exclusion.

In total, 219 patients met the inclusion criteria for the study: 135 (61.6%) were classified as REM and 84 (38.4%) were classified as n-REM (Fig. 1). Most of the REM patients identified as Hispanic (39.7%), followed by Black or African American (13.7%) and Asian (5.0%).

The median age of REM patients was significantly younger than that of n-REM patients (60 vs 69 years; *P* = .019). Additionally, a higher proportion of REM patients were enrolled in Medicaid compared to n-REM patients (66.7% vs 40.5%; *P* < .001).

Compared to n-REM patients, REM patients were significantly more likely to have type 2 diabetes mellitus (48.9% vs 23.8%; *P* < .001) and CKD (48.9% vs 21.4%; *P* < .001). No other significant differences were seen within recorded baseline comorbidities. Most patients (88.9%) were exposed to at least 1 antibiotic prior to their index CDI episode.

### CDI outcome

No significant differences were observed in the frequency of severe disease (40% vs 38.1%; *P* = .779) or fulminant disease (21.5% vs 11.9%; *P* = .072) between REM and n-REM patients, respectively. Among patients with fulminant disease, REM patients had numerically more cases of imaging-confirmed megacolon (27.6% vs 10%; *P* = .158) and CDI-associated surgical intervention (4.4% vs 1.2%; *P* = .183). Treatment for initial CDI in our study did not differ between REM versus n-REM patients, with most patients receiving oral vancomycin as a part of their regimen.

The results of the completed mediation analysis for the role of CKD as a mediator in the association between race or ethnicity and severe–fulminant CDI, while accounting for age, are shown in Table 1. The adjusted odds ratio reflecting the direct effect of race or ethnicity on severe or fulminant CDI was 1.56 (95% confidence interval [CI], 0.63–2.50). The adjusted odds ratio for the indirect effect mediated by CKD was 1.04 (95% CI, 0.81–1.27), and the adjusted odds ratio for the total effect was 1.63 (95% CI, 0.71–2.54), with 9.9% of the total effect mediated by CKD.

**Table 1.** Results

Variable	Overall, n/N (%)^a^	REM, n/N (%)^a^	N-REM, n/N (%)^a^	*P* Value
**Demographics**				
Total	219	135/219 (61.6)	84/219 (38.4)	…
Hispanic		87/219 (39.7)		
Black/African American		30/219 (13.7)		
Asian		11/219 (5.0)		
More than One Ethnicity		6/219 (2.7)		
Pacific Islander/Native Hawaiian		1/219 (0.5)		
Age, median y	62	60	69	0.019
Sex, male	108/219 (49.3)	62/135 (45.9)	46/84 (41.7)	0.203
Medicaid	124/219 (56.6)	90/135 (66.7)	34/84 (40.5)	<0.001
Hospital-onset CDI	133/219 (60.7)	85/135 (62.9)	48/84 (57.1)	0.479
**Clinical outcome**				
**CDI severity**				
Nonsevere	94/219 (42.9)	52/135 (38.5)	42/84 (50.0)	0.095
Severe	86/219 (39.3)	54/135 (40.0)	32/84 (38.1)	0.779
Fulminant	39/219 (17.8)	29/135 (21.5)	10/84 (11.9)	0.072
**Length of stay**				
LOS, median d	10	12	7	0.023
LOS >10 d	106/219 (48.4)	100/135 (74.0)	27/84 (32.0)	0.016
ICU LOS, median d	8	9	6	0.364
**Other clinical outcomes**				
All-cause ICU admission	79/219 (36.1)	57/135 (42.2)	22/84 (26.2)	0.016
Ileus	15/39 (38.5)	10/29 (34.5)	5/10 (50.0)	0.788
Megacolon	9/39 (23.1)	8/29 (27.6)	1/10 (10.0)	0.158
All-cause mortality during admission	25/219 (11.4)	14/135 (10.4)	11/84 (13.1)	0.537
CDI-associated surgical intervention	7/219 (3.2)	6/135 (4.4)	1/84 (1.2)	0.183
Diarrhea present after treatment	32/219 (14.6)	23/135 (17.0)	9/84 (10.7)	0.198
Recurrence	22/219 (10.0)	14/135 (10.4)	8/84 (9.5)	0.839
Patients who received oral vancomycin as a part of their regimen	218/219 (99.5)	134/135 (99.3)	84/84 (100)	1.0
Patients who received oral vancomycin and IV metronidazole as a part of their regimen	66/219 (30.1)	43/66 (65.2)	23/66 (34.8)	0.547
**Specialist consultation**				
ID consultation	63/219 (28.8)	45/135 (33.3)	18/84 (21.4)	0.058
GI consultation	28/219 (12.8)	21/135 (15.6)	7/84 (8.3)	0.120
**Comorbid conditions and risk factors**	Overall	REM	N-REM	*P* Value
**Comorbid conditions**				
Cardiovascular disease^b^	104/219 (47.5)	62/135 (45.9)	42/84 (50.0)	.557
Liver disease	22/219 (10.0)	13/135 (9.6)	9/84 (10.7)	.795
Diabetes	86/219 (39.3)	66/135 (48.9)	20/84 (23.8)	<.001
Chronic kidney disease	84/219 (38.4)	66/135 (48.9)	18/84 (21.4)	<.001
Cancer	35/219 (16.0)	23/135 (17.0)	12/84 (14.3)	.589
Charlson comorbidity index, median	5	5	5	.352
**CDI risk factors**				
Received any antibiotics prior to CDI	194/219 (88.9)	121/135 (89.6)	73/84 (86.9)	.537
No. of antibiotics used prior to admission prior to CDI therapy, median	0	0	0	.99
No. of antibiotics used during admission prior to CDI therapy, median	2.0	2	2	.99
Acid-reducing agent^c^	131/219 (59.8)	81/135 (60.0)	50/84 (59.5)	.944
Proton-pump inhibitor	86/219 (39.3)	52/135 (38.5)	34/84 (40.5)	.773
Immunosuppression^d^	50/219 (22.8)	34/135 (25.2)	16/84 (19.0)	.293
**Mediation analyses^e^**	Adjusted Odds Ratio (95% CI)			% Mediated
Total effect	1.62 (0.71–2.54)			
Direct effect	1.56 (0.63–2.50)			
Indirect effect	1.04 (0.81–1.27)			9.9

Note. CDI, *Clostridioides difficile* infection; CI, confidence interval; REM, racially and ethnically minoritized; N-REM, non–racially and ethnically minoritized; LOS, length of stay; ICU, intensive care unit; ID, infectious disease; GI, gastrointestinal; HIV, human immunodeficiency virus.

a Data are no. (%) unless otherwise specified.

b The patient was deemed to have cardiovascular disease if they had a history of 1 or more of the following in their electronic medical record: myocardial infarction, chronic heart failure, peripheral vascular disease, stroke, and/or transient ischemic attack in their electronic medical record.

c Acid reducing agents included antacids, histamine-2 receptor antagonists, and proton pump inhibitors.

d The patient was deemed to be immunosuppressed if they met 1 or more of the following criteria: takes immunosuppressants or 20 mg/d prednisone equivalence of corticosteroids, receiving active chemotherapy, HIV positive with CD4 <200.

e The association between race or ethnicity and severe or fulminant *Clostridioides difficile* infection and the role of chronic kidney disease as a mediator

The overall median LOS was significantly longer in the REM group compared to the n-REM group (12 vs 7 days; *P* = .023), and patients in the REM group were significantly more likely to have a LOS extend beyond 10 days (74% vs 32%; *P* = .016). REM patients were also more likely to have all-cause ICU admission (42.2% vs 26.2%; *P* = .016), and if admitted, had longer median ICU LOS (9 vs 6 days; *P* = .364), though not statistically significant. Despite differences in morbidity, there were no significant differences in CDI recurrence (10.4% vs. 9.5%; *P* = .839) or all-cause mortality (10.4% vs 13.1%; *P* = .537). REM patients received more ID (33.3% vs 21.4%; *P* = .058) and GI consultations (15.6% vs 8.3%; *P* = .12) compared with n-REM patients, but these differences were not significant.

## Discussion

In this study, we observed trends in worse clinical outcomes in REM patients compared to n-REM patients diagnosed with initial CDI. Despite a nearly 10-year separation in the median age recorded, REM patients were more likely to require an ICU stay and to have a prolonged hospitalization, though this may not be solely attributable to CDI. This finding is contrary to the established understanding that older age is a risk factor for acquiring CDI and having worse clinical outcomes.^5^ Nonetheless, the additional findings align with other published studies reporting that Black patients had longer hospital LOS and more severe CDI.^4,8^ We also observed a higher incidence of ID and GI consultation for REM patients, likely due to the higher incidence of severe and fulminant CDI. Nevertheless, we did not detect a significant difference according to the treatment received. Although the cost incurred during admission was not analyzed in our study, the association between longer hospital LOS and higher cost for both the patient and hospital have been reported in prior studies.^8,9^

The REM patients in our study were also more likely to have type 2 diabetes and CKD. Of interest, type 2 diabetes is a leading cause of CKD in the United States, and the prevalence of chronic diseases (including type 2 diabetes) is higher in REM groups than in n-REM individuals.^6,10^ Although not statistically significant, our mediation analysis suggested that REM is associated with 1.63 increased odds of severe or fulminant CDI compared to n-REM, and ∼10% of this increase is mediated by pre-existing CKD.

The potential relationship between medical insurance type or status, which is closely linked to socioeconomic standing, and CDI outcomes has been demonstrated previously.^9^ Patients with Medicaid treated for CDI have a 16% higher readmission rate compared to those with Medicare.^9^ Although we did not assess the direct impact of medical insurance on clinical outcomes, descriptively, REM patients were more likely to have Medicaid as their primary insurance, further demonstrating the probable influence of insurance type (or socioeconomic status) on deleterious health outcomes.

Our findings shed light on the potential impact of inequities in social determinants of health, at the intersection of race or ethnicity, on initial CDI outcomes. Nevertheless, this study had several limitations. Due to the retrospective nature of this study, we were unable to capture patient data not reported, which includes the timing of symptom onset to patient diagnosis. Also, several clinical outcomes including recurrence rate and antibiotic use prior to admission could not be assessed if they occurred outside our institution. In addition, we did not collect additional information to assess the patient’s social vulnerability beyond the patient’s insurance status. Furthermore, because CKD was the only variable explored as a mediator, other potential mediators were not included in this analysis and should be considered for future studies. Lastly, the conclusions from this study are limited by our definition of REM versus n-REM. In summary, further research is needed to continue to address health-equity gaps in infectious diseases.

## Supplementary material

For supplementary material accompanying this paper visit https://doi.org/10.1017/ice.2023.84. click here to view supplementary material
