# Supplementary figures and images for: Examining the impact of racial disparities on Clostridioides difficile infection outcomes at a Southern California academic teaching hospital
Source: Infect Control Hosp Epidemiol. 2023 May 4;44(11):1861–5. doi: 10.1017/ice.2023.84 (PMC10665859; doi:10.1017/ice.2023.84)

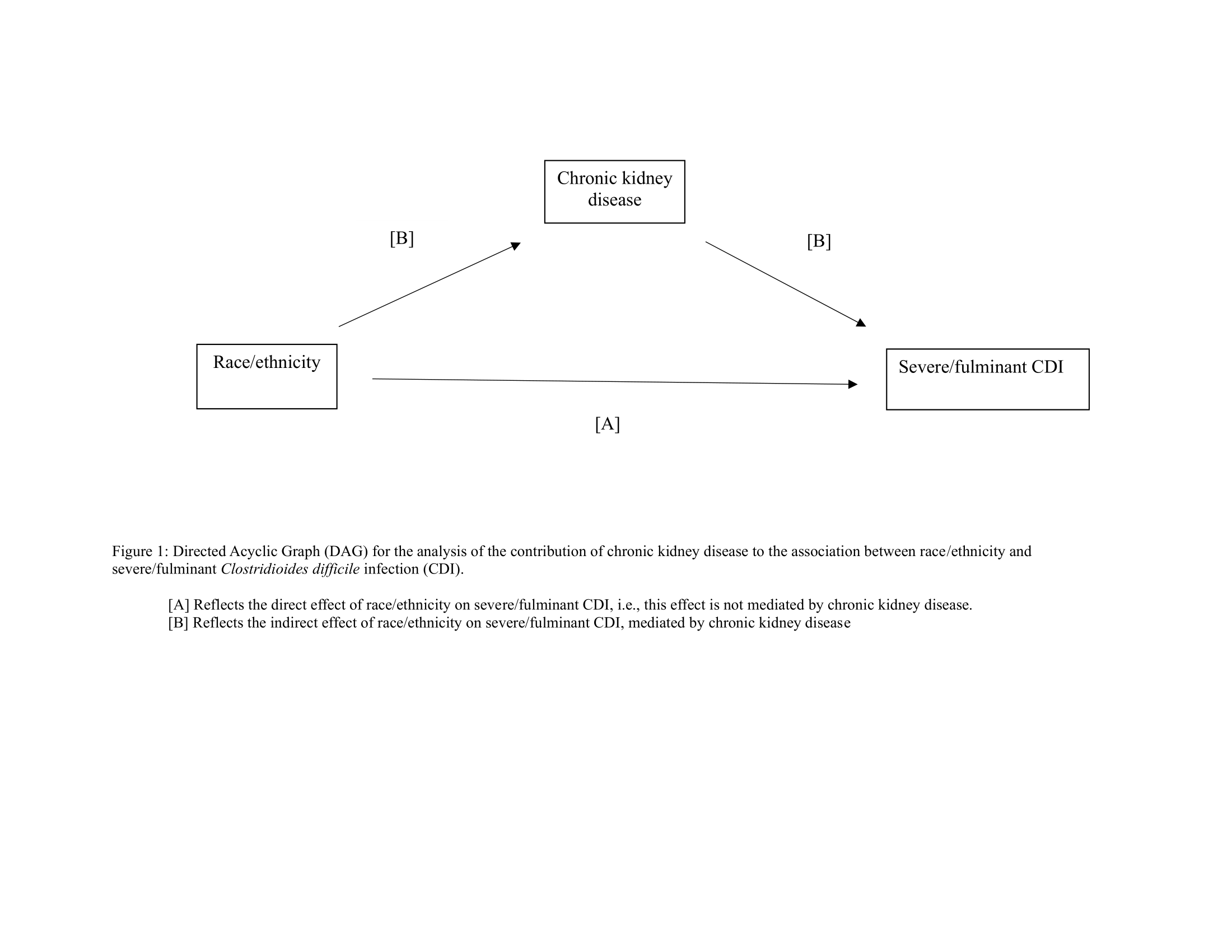

Supplement: Supplementary file 1 [file S0899823X23000843sup001.tiff]
